# Supplementary material for: Curated and harmonised transcriptomics datasets of interstitial lung diseases
Source: Data Brief. 2025 Oct 14;63:112139. doi: 10.1016/j.dib.2025.112139 (PMC12581653; doi:10.1016/j.dib.2025.112139)
Supplement: Supplementary file 1 [file mmc1.zip › Supplementary_material/RNA-seq/fastQC reports/GSE184316/TRIMMED_SRR15926756_fastqc.html]

TRIMMED\_SRR15926756.fastq.gz FastQC Report 

FastQC Report

Sat 3 Dec 2022  
TRIMMED\_SRR15926756.fastq.gz

## Summary

- Basic Statistics
- Per base sequence quality
- Per sequence quality scores
- Per base sequence content
- Per sequence GC content
- Per base N content
- Sequence Length Distribution
- Sequence Duplication Levels
- Overrepresented sequences
- Adapter Content

## Basic Statistics

| Measure | Value |
| --- | --- |
| Filename | TRIMMED\_SRR15926756.fastq.gz |
| File type | Conventional base calls |
| Encoding | Sanger / Illumina 1.9 |
| Total Sequences | 7461197 |
| Sequences flagged as poor quality | 0 |
| Sequence length | 35-371 |
| %GC | 52 |

## Per base sequence quality

## Per sequence quality scores

## Per base sequence content

## Per sequence GC content

## Per base N content

## Sequence Length Distribution

## Sequence Duplication Levels

## Overrepresented sequences

| Sequence | Count | Percentage | Possible Source |
| --- | --- | --- | --- |
| CAGTGGGGCCACGAGCTGAGTGCGTCCTGTCACTCCACTCCCATGTCCCT | 71176 | 0.9539488100903916 | No Hit |
| CACCTCCCCGAAGCTCAGGGAGAGCCCTGTTAGGGCCGCCTCTGGCCCTA | 63111 | 0.8458562345961379 | No Hit |
| AGGCTACACACTGAGGACTCTGTTCCTCCCCTTTCCGCCTAGGGGAAAGT | 43914 | 0.5885650787668519 | No Hit |
| GGGAAGCGGGGAATGTCTACGTGCGTATGCACGTGGCACTCTCTGCCCGA | 33658 | 0.45110724190769924 | No Hit |
| TGATATGGTTCCTGGCAAGCCCATGTGTGTTGAGAGCTTCTCAGACTATC | 29802 | 0.39942652633350917 | No Hit |
| CAATCCACTTGATCCCAACTCATCTCTCATTTATTTCGGCTTCTTTTATT | 29355 | 0.39343553051876257 | No Hit |
| TTACAAAAGCAGAATAAAAGCGAAAAGAAATGAAAATGTTACACTACATT | 28243 | 0.37853175569550035 | No Hit |
| TGATGACACCCACCGCAACTGTCTGTCTCATATCACGAACAGCAAAGCGA | 27517 | 0.3688014134997374 | No Hit |
| TACCACTGCCACCTTCTCCATCGGCTCCACTGGCCTCGTGGTGTATGACT | 25633 | 0.3435507734214765 | No Hit |
| CTTCATGATGTAGCAGCAGGTGCCAGGGGCTGGCTTGTAGGCGATCAGCA | 25015 | 0.33526792014739726 | No Hit |
| GGCAATGAGCGGTTCCGCTGCCCTGAGGCACTCTTCCAGCCTTCCTTCCT | 18965 | 0.2541817351827059 | No Hit |
| GTCCACGTCACACTTCATGATGGAGTTGAAGGTAGTTTCGTGGATGCCAC | 16258 | 0.21790069341420687 | No Hit |
| GGCATCAAAAGTGATGGACTGCCCATTGCTGGAGAAGACCTTCTCTCCTA | 15931 | 0.2135180186235533 | No Hit |
| AGACATCAAATCCTGCAGACAAGGGGAGCCCTCAGTCTGCAGGGCTCCAT | 15486 | 0.20755382815920823 | No Hit |
| AGAAGTCACTGATCAGACACACTAGTGTGGCCTTGTTGGCTTGGAGCTCC | 15078 | 0.20208553667729187 | No Hit |
| TTCAGCCCTGATCAAGACATGAGGGAGGCAGGGGCTCAGCTGAAGAAGCT | 14739 | 0.19754202978422897 | No Hit |
| CAATGGGGTACTTCAGGGTCAGGATGCCACGCTTGCTCTGGGCCTCGTCG | 13262 | 0.17774627851268368 | No Hit |
| CTCTCTTTCTGGCCTGGAGGCTATCCAGCGTACTCCAAAGATTCAGGTTT | 13224 | 0.1772369768550542 | No Hit |
| AGCTGCTAAGTGCTGTGTTGTCGTTCCCCCTGCTTAAAATAAAGTTGTTT | 12971 | 0.17384610002925804 | No Hit |
| ACCTCTCTCTGGGCTTCTATTTCGACCGCGATGATGTGGCTCTGGAAGGC | 12738 | 0.17072327670747736 | No Hit |
| CCCTGGCTGCTACAGGAGAATAGCAGACAGGTATAGTTAAGAAACAACTT | 12571 | 0.16848502994894787 | No Hit |
| ACAAGAAGTACATCAAAGATTACATGAAATCAATCAAAGGGAAACTTGAA | 12527 | 0.16789531224011375 | No Hit |
| GAAACCCAGACACATAGCAATTCAGGAAATTTGACTTTCCATTCTCTGCT | 12251 | 0.16419617388469973 | No Hit |
| ACGTTTACCTGTCCATGTCTTACTACTTTGACCGCGATGATGTGGCTTTG | 10356 | 0.1387981043792303 | No Hit |
| CAGCAGTTTACACCTGCTAGGGTGTTCAAAGGTCAGTGCTATAGAAATTC | 10227 | 0.13706915927833027 | No Hit |
| ATCAAAGAAGAACATGTGATCATCCAGGCCGAGTTCTATCTGAATCCTGA | 9858 | 0.13212357212924414 | No Hit |
| CAGCATGTTCCCTCTCCTCATGAGATTGGTGAAGAAAGTATTTGGCAAAG | 9813 | 0.13152045174520924 | No Hit |
| GGCAATGAGCGGTTCCGGTGTCCGGAGGCGCTGTTCCAGCCTTCCTTCCT | 9196 | 0.12325100114633082 | No Hit |
| GTCCACGTCACACTTCATGATGGAGTTGAAGGTGGTCTCGTGGATGCCGC | 9107 | 0.12205816305346179 | No Hit |
| GTTCTGCAGCCCCTGTCATAAAAGGTTTTACTCTTTCTGGTCTCTGTTCT | 9106 | 0.12204476037826102 | No Hit |
| AACAAGCACAAAGCCAAGAAAACCAACGATGCCAGATACTGAATTTCTAT | 9079 | 0.12168288814784008 | No Hit |
| CGATCAGCACGGGCTCGTACTCTGCCACAAACTGATCACACTGCTTCTGG | 8781 | 0.11768889093800902 | No Hit |
| GGGACCAAGGTCACCGTCCTAGGTCAGCCCAAGGCCAACCCCACTGTCAC | 7976 | 0.10689973740138481 | No Hit |
| CTCCGCCGCCTCCGCCGCAGACGCCGCCGCGATGCGCTACGTCGCCTCCT | 7750 | 0.10387073280600954 | No Hit |
| GGAAGACAGTGTGGCGATTCCTCAGGGATCTAGAACTAGAAATACCATTG | 7497 | 0.10047985598021336 | No Hit |

## Adapter Content

Produced by FastQC (version 0.11.7)
